# Impact of Vedic Chants Intervention Programme on Autistic Spectrum Disorder

**DOI:** 10.1186/1755-8166-7-S1-P129

**Published:** 2014-01-21

**Authors:** Kandasamy Dinesh Kumar, Sushruth Badhe, Sathiyavedu Santhiya

**Affiliations:** 1 Dept of Genetics, Dr ALM PG IBMS, University of Madras, Taramani Campus, Chennai, India; 2 Midham charitable trust, Puduchery, India

## Background

Autism Spectrum Disorder (ASD) is a developmental disorder that affects the behavior and social communication of the child. In India, awareness about Autism coupled with shortage of skilled professionals poses severe constraints in management of children with ASD. There arises a need to explore the avenues of alternate therapy in the form of Group Therapy. A 44-bp insertion/deletion polymorphism in the promoter region of the serotonin receptor gene *5-HTT* gene (5-HTTLPR) has been identified to modify the transcription rate of the gene (Lesch et al., 1996; Greenberg et al., 1999). The short allelic variant has been shown to reduce *5-HTT* transcription resulting in diminished 5-HTT levels and reduced serotonin (*5-HTT*) reuptake into human platelets. We speculate the possibility of altered hormone levels (Melatonin and Serotonin) in individuals with ASD.

As per earlier reports an association between 5-HTT gene polymorphism and autism has been indicated. Recent evidences from clinical and neuroscience research suggests an approach based on yogic principles and meditative tools is worth experimenting in children with autism.

The same could be addressed in ASD individuals to ameliorate the drastic effect of these hormones on ASD individuals.

## Methods

A single group pre-/ post- test study design was employed. With the aid of a social and behavioral assessment scale for ASD, fifteen children with ASD between the age group of 7 - 14 years were evaluated for assessing the effect of the Vedic Chants Intervention Program recruited from special schools in and around Chennai. Chanting of select Sanskrit Mantras by an experienced meditator were given as an auditory stimulus in a closed room to the children for a period of 20 min daily at a fixed time. Alterations in the endocrine profile (Melatonin and Serotonin) were measured using ELISA Kits. Polymorphism in serotonin transporter (*5-HTT*) is to be analyzed to correlate the endocrine findings.

## Results

Study shows that there is a difference in the pre & post-test mean achievement scores due to the Vedic Chants Intervention Program. Some of the qualitative changes observed by the special educators were a reduction in the hyper activity, self-biting and other common traits of ASD like head nodding, hand flapping etc. This reflected in their class room sitting as well. Some of the children were remembering certain lines of the mantras and imitating the sound ‘Om’ and responding to the ‘Namaste’ gesture. Most of the children including those with ADHD sat throughout the entire session. The endocrine profile of the subjects was altered.

## Conclusions

The auditory stimulus has the potential to provoke the cognitive abilities of the children. Vedic Chants therapy might be one of the efficient Group therapies for the management of children with ASD.

